# A second monoclinic polymorph of {2,6-bis[(2,4,5-trifluoro­phen­yl)imino­meth­yl]pyridine-κ^3^ *N*,*N*′,*N*′′}dichloridonickel(II)

**DOI:** 10.1107/S1600536811055577

**Published:** 2012-01-11

**Authors:** Oscar Baldovino-Pantaleón, Simón Hernández-Ortega, Reyna Reyes-Martínez, David Morales-Morales

**Affiliations:** a UAM Reynosa Rodhe, Universidad Autónoma de Tamaulipas, Carr. Reynosa-San Fernando S/N, Reynosa, Tamaulipas 88779, Mexico; b Instituto de Química, Universidad Nacional Autónoma de México, Circuito Exterior, Ciudad Universitaria, México, DF, 04510, Mexico

## Abstract

The asymmetric unit of the title compound, [NiCl_2_(C_19_H_9_F_6_N_3_)], contains one half-mol­ecule residing on a crystallographic twofold rotation axis. The title compound crystallizes in space group *C*2/*c* while the previously reported polymorph was reported in *P*2_1_/*c* [Baldovino-Pantaleón *et al.* (2006 ▶). *Adv. Synth. Catal.* **348**, 236–242]. The Ni^2+^ ion exhibits a penta­coordinate distorted trigonal–bipyramidal NiCl_2_N_3_ geometry, with two Cl atoms in the equatorial plane. In the crystal, mol­ecules are linked by inter­molecular C—F⋯π [F⋯centroid = 2.9676 (14) Å] inter­actions.

## Related literature

For related studies, see: Baldovino-Pantaleón *et al.* (2005 ▶, 2006 ▶); Morales-Morales (2008 ▶); Serrano-Becerra & Morales-Morales (2009 ▶). For catalysis reactions, see: Gómez-Benítez *et al.* (2006 ▶). For the previously reported polymorph, see; Baldovino-Pantaleón *et al.* (2006 ▶).
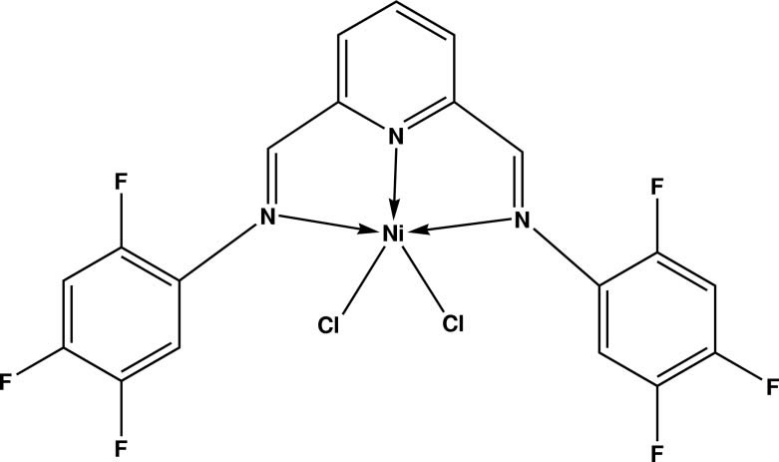


## Experimental

### 

#### Crystal data

[NiCl_2_(C_19_H_9_F_6_N_3_)]
*M* *_r_* = 522.90
Monoclinic, 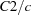

*a* = 18.0947 (13) Å
*b* = 8.8967 (6) Å
*c* = 12.1638 (9) Å
β = 101.421 (2)°
*V* = 1919.4 (2) Å^3^
*Z* = 4
Mo *K*α radiation
μ = 1.36 mm^−1^
*T* = 298 K
0.32 × 0.16 × 0.06 mm

#### Data collection

Bruker SMART APEX CCD area-detector diffractometer
Absorption correction: analytical (*SHELXTL*; Sheldrick, 2008 ▶) *T*_min_ = 0.740, *T*_max_ = 0.921
7821 measured reflections
1751 independent reflections
1482 reflections with *I* > 2σ(*I*)
*R*_int_ = 0.033

#### Refinement

*R*[*F* ^2^ > 2σ(*F* ^2^)] = 0.025
*wR*(*F* ^2^) = 0.058
*S* = 0.96
1751 reflections
142 parameters
H-atom parameters constrained
Δρ_max_ = 0.23 e Å^−3^
Δρ_min_ = −0.20 e Å^−3^

### 

Data collection: *SMART* (Bruker, 2007 ▶); cell refinement: *SAINT* (Bruker, 2007 ▶); data reduction: *SAINT*; program(s) used to solve structure: *SHELXS97* (Sheldrick, 2008 ▶); program(s) used to refine structure: *SHELXL97* (Sheldrick, 2008 ▶); molecular graphics: *SHELXTL* (Sheldrick, 2008 ▶); software used to prepare material for publication: *SHELXTL*.

## Supplementary Material

Crystal structure: contains datablock(s) I, global. DOI: 10.1107/S1600536811055577/fj2491sup1.cif

Structure factors: contains datablock(s) I. DOI: 10.1107/S1600536811055577/fj2491Isup2.hkl

Additional supplementary materials:  crystallographic information; 3D view; checkCIF report

## Figures and Tables

**Fig. 1.**
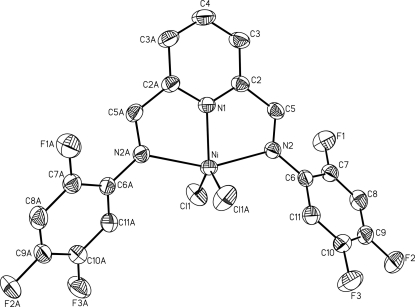
The molecular structure of I with the numbering scheme. Displacement ellipsoids are shown at the 40% probability level. H atoms have been omitted for clarity.

## supplementary crystallographic information

### Comment

For the last decade our research group has focused on the design, synthesis and use of pincer-type ligands and their transition metal complexes for novel organic transformations (Morales-Morales 2008; Serrano-Becerra & Morales-Morales, 2009). The selection of these ligands has been done on the basis of the robustness that they confer to the transition metal complexes they form (Baldovino-Pantaleón, *et al.*, 2005, 2006). In recent years this kind of complexes have gained more interest particularly those derived from Ni, due to the importance that cross-coupling reactions have gained in important organic transformations of potential industrial relevance and the potential application of the nickel derivatives for the same sort of process regularly catalized by analogous palladium derivatives, but using a far more cheaper metal (Gómez-Benítez *et al.*, 2006). The title compound is a polymorphous of a compound described previously (Baldovino-Pantaleón, *et al.*, 2006).

The molecular structure of I is shown in figure 1 with the numbering scheme. In comparison, compound II described previously (Baldovino-Pantaleón, *et al.*, 2006), was crystallized in a monoclinic (P 2_1_/c), while compound I, crystallizes in space group C 2/c. The asymmetric unit consists of a half molecule with Ni—N1—C4—H4 in a special position (1/2, *y*, 1/4), and by two fold axis the complete molecule is generated. The Ni atom is found in a pentacoordinated distorted bipyramidal geometry with the two chlorides occupying the equatorial positions. While the fragment N1—Ni—N2—N2A is planar, the pyridine ring is slightly rotated by 5.4 (1)°. The 2,4,5-trifluorophenyl ring is not coplanar and is forming a dihedral angle of 39.41 (5)° with the coordination metallic center, while in II, the fluorophenyl rings have dihedral angles of 14.4 (2)° and 13.4 (2)° with the coordination metallic center. The bond distances Ni—*N*(imino) in I are slightly shorter than in II. In absence of clasic aceptor-donor H atom, the weak interactions become very important stabilizing the crystal structure. The molecules in the crystal structure are linked by C—H—F—C, C—H—π, C—F—π and C—F—F—C intermolecular interactions (Table 1).

### Experimental

A solution of the ligand {C_5_H_3_N-2,6-(CH=N—C_6_H_2_-2,4,5-F_3_)_2_} (120 mg, 0.33 mmol) in anhydrous CH_2_Cl_2_ (10 ml) was added to a stirred solution of NiCl_2_.6 H_2_O(0.078 g, 0.33 mmol) in absolute methanol (10 ml). The resulting green solution was stirred at room temperature for 2 h. After this time a red suspension is noted and the solvent evaporated under vacuum. The product was purified by recrystallization from MeOH; the resulting precipitated was filtered and washed with hexane (3 *X* 5 ml) and dried under vacuum. Crystals suitable for single-crystal X-ray diffraction studies were obtained from a dichloromethane/ methanol (4:1) solution. A red solid was obtained; yield: 142 mg (88%); mp: 240°C

### Refinement

H atoms were included in calculated positions (C—H = 0.93 Å), and refined using a riding model,with *U*_iso_(H) = 1.2*U*_eq_ of the carrier atom.

### Crystal data

**Table:**

[NiCl_2_(C_19_H_9_F_6_N_3_)]	*F*(000) = 1040
*M**_r_* = 522.90	*D*_x_ = 1.810 Mg m^−^^3^
Monoclinic, *C*2/*c*	Mo *K*α radiation, λ = 0.71073 Å
Hall symbol: -C 2yc	Cell parameters from 5009 reflections
*a* = 18.0947 (13) Å	θ = 2.6–32.1°
*b* = 8.8967 (6) Å	µ = 1.36 mm^−^^1^
*c* = 12.1638 (9) Å	*T* = 298 K
β = 101.421 (2)°	Prism, red
*V* = 1919.4 (2) Å^3^	0.32 × 0.16 × 0.06 mm
*Z* = 4	

### Data collection

**Table:**

Bruker SMART APEX CCD area-detector diffractometer	1751 independent reflections
Radiation source: fine-focus sealed tube	1482 reflections with *I* > 2σ(*I*)
graphite	*R*_int_ = 0.033
Detector resolution: 0.83 pixels mm^-1^	θ_max_ = 25.3°, θ_min_ = 2.3°
ω scans	*h* = −21→21
Absorption correction: analytical (*SHELXTL*; Sheldrick, 2008)	*k* = −10→10
*T*_min_ = 0.740, *T*_max_ = 0.921	*l* = −14→14
7821 measured reflections	

### Refinement

**Table:**

Refinement on *F*^2^	Primary atom site location: structure-invariant direct methods
Least-squares matrix: full	Secondary atom site location: difference Fourier map
*R*[*F*^2^ > 2σ(*F*^2^)] = 0.025	Hydrogen site location: inferred from neighbouring sites
*wR*(*F*^2^) = 0.058	H-atom parameters constrained
*S* = 0.96	*w* = 1/[σ^2^(*F*_o_^2^) + (0.034*P*)^2^] where *P* = (*F*_o_^2^ + 2*F*_c_^2^)/3
1751 reflections	(Δ/σ)_max_ < 0.001
142 parameters	Δρ_max_ = 0.23 e Å^−^^3^
0 restraints	Δρ_min_ = −0.20 e Å^−^^3^

### Special details

**Table:**

Geometry. All e.s.d.'s (except the e.s.d. in the dihedral angle between two l.s. planes) are estimated using the full covariance matrix. The cell e.s.d.'s are taken into account individually in the estimation of e.s.d.'s in distances, angles and torsion angles; correlations between e.s.d.'s in cell parameters are only used when they are defined by crystal symmetry. An approximate (isotropic) treatment of cell e.s.d.'s is used for estimating e.s.d.'s involving l.s. planes.
Refinement. Refinement of *F*^2^ against ALL reflections. The weighted *R*-factor *wR* and goodness of fit *S* are based on *F*^2^, conventional *R*-factors *R* are based on *F*, with *F* set to zero for negative *F*^2^. The threshold expression of *F*^2^ > σ(*F*^2^) is used only for calculating *R*-factors(gt) *etc*. and is not relevant to the choice of reflections for refinement. *R*-factors based on *F*^2^ are statistically about twice as large as those based on *F*, and *R*- factors based on ALL data will be even larger.

### Fractional atomic coordinates and isotropic or equivalent isotropic displacement parameters (Å^2^)

**Table:**

	*x*	*y*	*z*	*U*_iso_*/*U*_eq_	
Ni1	0.5000	0.68998 (3)	0.2500	0.03615 (12)	
Cl1	0.42526 (3)	0.79023 (6)	0.35781 (4)	0.06007 (17)	
F1	0.62550 (7)	0.60471 (13)	0.63576 (9)	0.0642 (3)	
F2	0.75608 (7)	1.06173 (13)	0.66763 (10)	0.0688 (4)	
F3	0.72148 (8)	1.09768 (14)	0.44569 (11)	0.0767 (4)	
N1	0.5000	0.4707 (2)	0.2500	0.0352 (5)	
N2	0.58620 (8)	0.63599 (16)	0.39784 (11)	0.0372 (3)	
C2	0.54313 (10)	0.39674 (18)	0.33462 (14)	0.0384 (4)	
C3	0.54303 (12)	0.2412 (2)	0.33833 (16)	0.0505 (5)	
H3	0.5714	0.1902	0.3991	0.061*	
C4	0.5000	0.1634 (3)	0.2500	0.0586 (8)	
H4	0.5000	0.0588	0.2500	0.070*	
C5	0.58923 (10)	0.4948 (2)	0.41731 (13)	0.0399 (4)	
H5	0.6196	0.4559	0.4817	0.048*	
C6	0.63018 (9)	0.7410 (2)	0.47162 (14)	0.0376 (4)	
C7	0.64917 (10)	0.7265 (2)	0.58667 (15)	0.0428 (4)	
C8	0.69027 (11)	0.8327 (2)	0.65465 (16)	0.0513 (5)	
H8	0.7015	0.8207	0.7321	0.062*	
C9	0.71419 (10)	0.9570 (2)	0.60497 (16)	0.0478 (5)	
C10	0.69631 (11)	0.9745 (2)	0.49109 (16)	0.0483 (5)	
C11	0.65417 (10)	0.8698 (2)	0.42393 (15)	0.0461 (5)	
H11	0.6416	0.8847	0.3468	0.055*	

### Atomic displacement parameters (Å^2^)

**Table:**

	*U*^11^	*U*^22^	*U*^33^	*U*^12^	*U*^13^	*U*^23^
Ni1	0.0457 (2)	0.02916 (18)	0.03209 (19)	0.000	0.00412 (14)	0.000
Cl1	0.0713 (4)	0.0718 (4)	0.0386 (3)	0.0258 (3)	0.0145 (2)	0.0021 (2)
F1	0.0917 (9)	0.0633 (8)	0.0368 (6)	−0.0145 (7)	0.0106 (6)	0.0031 (5)
F2	0.0692 (8)	0.0631 (8)	0.0648 (8)	−0.0106 (6)	−0.0090 (6)	−0.0235 (6)
F3	0.0997 (10)	0.0570 (8)	0.0663 (8)	−0.0309 (7)	−0.0009 (7)	0.0030 (6)
N1	0.0434 (12)	0.0312 (10)	0.0307 (10)	0.000	0.0065 (9)	0.000
N2	0.0413 (8)	0.0373 (8)	0.0316 (8)	0.0031 (7)	0.0036 (6)	−0.0016 (6)
C2	0.0476 (10)	0.0339 (10)	0.0336 (9)	0.0044 (8)	0.0077 (8)	0.0027 (7)
C3	0.0710 (14)	0.0346 (10)	0.0429 (11)	0.0078 (10)	0.0041 (10)	0.0056 (8)
C4	0.088 (2)	0.0300 (14)	0.0558 (18)	0.000	0.0083 (16)	0.000
C5	0.0465 (10)	0.0387 (10)	0.0320 (9)	0.0088 (8)	0.0013 (8)	0.0018 (8)
C6	0.0361 (10)	0.0380 (9)	0.0360 (9)	0.0060 (8)	0.0004 (8)	−0.0033 (8)
C7	0.0464 (11)	0.0434 (11)	0.0372 (10)	0.0040 (8)	0.0051 (8)	−0.0018 (8)
C8	0.0555 (12)	0.0616 (13)	0.0332 (10)	0.0073 (10)	−0.0002 (9)	−0.0080 (9)
C9	0.0424 (11)	0.0463 (12)	0.0501 (11)	0.0038 (9)	−0.0017 (9)	−0.0147 (9)
C10	0.0491 (11)	0.0427 (11)	0.0499 (12)	−0.0027 (9)	0.0022 (9)	−0.0009 (9)
C11	0.0517 (11)	0.0448 (11)	0.0372 (10)	0.0001 (9)	−0.0018 (8)	0.0006 (8)

### Geometric parameters (Å, °)

**Table:**

Ni1—N1	1.9508 (19)	C3—C4	1.382 (2)
Ni1—N2^i^	2.1878 (13)	C3—H3	0.9300
Ni1—N2	2.1878 (13)	C4—C3^i^	1.382 (2)
Ni1—Cl1^i^	2.2464 (5)	C4—H4	0.9300
Ni1—Cl1	2.2464 (5)	C5—H5	0.9300
F1—C7	1.347 (2)	C6—C7	1.379 (2)
F2—C9	1.3400 (19)	C6—C11	1.393 (3)
F3—C10	1.347 (2)	C7—C8	1.374 (3)
N1—C2^i^	1.3354 (18)	C8—C9	1.370 (3)
N1—C2	1.3354 (18)	C8—H8	0.9300
N2—C5	1.277 (2)	C9—C10	1.368 (3)
N2—C6	1.424 (2)	C10—C11	1.367 (3)
C2—C3	1.384 (3)	C11—H11	0.9300
C2—C5	1.462 (2)		
			
N1—Ni1—N2^i^	77.32 (4)	C3—C4—H4	120.1
N1—Ni1—N2	77.32 (4)	C3^i^—C4—H4	120.1
N2^i^—Ni1—N2	154.64 (8)	N2—C5—C2	117.50 (15)
N1—Ni1—Cl1^i^	113.393 (16)	N2—C5—H5	121.2
N2^i^—Ni1—Cl1^i^	91.20 (4)	C2—C5—H5	121.2
N2—Ni1—Cl1^i^	98.82 (4)	C7—C6—C11	117.64 (16)
N1—Ni1—Cl1	113.393 (16)	C7—C6—N2	125.03 (17)
N2^i^—Ni1—Cl1	98.82 (4)	C11—C6—N2	117.31 (16)
N2—Ni1—Cl1	91.20 (4)	F1—C7—C8	117.93 (17)
Cl1^i^—Ni1—Cl1	133.21 (3)	F1—C7—C6	119.18 (16)
C2^i^—N1—C2	121.0 (2)	C8—C7—C6	122.88 (18)
C2^i^—N1—Ni1	119.52 (10)	C9—C8—C7	118.05 (18)
C2—N1—Ni1	119.52 (10)	C9—C8—H8	121.0
C5—N2—C6	122.00 (15)	C7—C8—H8	121.0
C5—N2—Ni1	111.45 (11)	F2—C9—C10	119.38 (18)
C6—N2—Ni1	126.33 (11)	F2—C9—C8	120.18 (18)
N1—C2—C3	120.88 (17)	C10—C9—C8	120.44 (17)
N1—C2—C5	113.77 (15)	F3—C10—C11	120.20 (17)
C3—C2—C5	125.34 (16)	F3—C10—C9	118.48 (16)
C4—C3—C2	118.68 (18)	C11—C10—C9	121.32 (18)
C4—C3—H3	120.7	C10—C11—C6	119.65 (17)
C2—C3—H3	120.7	C10—C11—H11	120.2
C3—C4—C3^i^	119.8 (3)	C6—C11—H11	120.2
			
N2^i^—Ni1—N1—C2^i^	4.58 (9)	Ni1—N2—C5—C2	6.53 (19)
N2—Ni1—N1—C2^i^	−175.42 (9)	N1—C2—C5—N2	−3.1 (2)
Cl1^i^—Ni1—N1—C2^i^	−81.18 (9)	C3—C2—C5—N2	175.64 (17)
Cl1—Ni1—N1—C2^i^	98.82 (9)	C5—N2—C6—C7	−33.8 (3)
N2^i^—Ni1—N1—C2	−175.42 (9)	Ni1—N2—C6—C7	140.38 (15)
N2—Ni1—N1—C2	4.58 (9)	C5—N2—C6—C11	147.89 (17)
Cl1^i^—Ni1—N1—C2	98.82 (9)	Ni1—N2—C6—C11	−37.9 (2)
Cl1—Ni1—N1—C2	−81.18 (9)	C11—C6—C7—F1	178.61 (16)
N1—Ni1—N2—C5	−6.01 (11)	N2—C6—C7—F1	0.3 (3)
N2^i^—Ni1—N2—C5	−6.01 (11)	C11—C6—C7—C8	−0.1 (3)
Cl1^i^—Ni1—N2—C5	−118.15 (11)	N2—C6—C7—C8	−178.39 (17)
Cl1—Ni1—N2—C5	107.72 (12)	F1—C7—C8—C9	−179.78 (17)
N1—Ni1—N2—C6	179.28 (14)	C6—C7—C8—C9	−1.1 (3)
N2^i^—Ni1—N2—C6	179.28 (14)	C7—C8—C9—F2	−178.30 (16)
Cl1^i^—Ni1—N2—C6	67.14 (13)	C7—C8—C9—C10	1.0 (3)
Cl1—Ni1—N2—C6	−66.99 (13)	F2—C9—C10—F3	−0.2 (3)
C2^i^—N1—C2—C3	−1.51 (13)	C8—C9—C10—F3	−179.49 (18)
Ni1—N1—C2—C3	178.49 (13)	F2—C9—C10—C11	179.53 (17)
C2^i^—N1—C2—C5	177.34 (16)	C8—C9—C10—C11	0.2 (3)
Ni1—N1—C2—C5	−2.66 (16)	F3—C10—C11—C6	178.29 (17)
N1—C2—C3—C4	3.0 (2)	C9—C10—C11—C6	−1.4 (3)
C5—C2—C3—C4	−175.74 (15)	C7—C6—C11—C10	1.3 (3)
C2—C3—C4—C3^i^	−1.44 (12)	N2—C6—C11—C10	179.76 (16)
C6—N2—C5—C2	−178.49 (15)		

Symmetry codes: (i) −*x*+1, *y*, −*z*+1/2.

### Table 1 Table 1. Intermolecular and intramolecular interaction C-F–π, C-H–π, π–π of (I) (Å)

**Table:**

H/F/centroid	centroid/F	distance	Symmetry-code
H8	F3	2.652	(i)
H8	F2	2.648	(ii)
F1	F2	2.903	(iii)
F1	N1-C4	2.968	(iv)

(i) x, 2-y, 1.5 +z; (ii) -x+1.5, +y-1/2', -z+1.5; (iii) -x+1.5, 1.5+y, -z+1.5; (iv) x, -y+1, z-1/2
